# Building integrated data systems for health and nutrition program evaluations: lessons learned from a multi-country implementation of a DHIS 2-based system

**DOI:** 10.7189/jogh.08.020307

**Published:** 2018-12

**Authors:** Elizabeth Hazel, Emily Wilson, Adebusoye Anifalaje, Talata Sawadogo-Lewis, Rebecca Heidkamp

**Affiliations:** 1 Department of International Health, Johns Hopkins Bloomberg School of Public Health, Baltimore, Maryland, USA; 2 BAO Systems, Georgetown, Washington, D.C., USA

Globally, we have more data on population health than ever before. In the era of “big data”, the challenge is not only to collect data but also to make use and sense of what is already there. Countries have a wealth of information on maternal, newborn and child health and nutrition (MNCH&N) programs and impact but it is dispersed through a variety of sources, and sometimes difficult for policy and program decision-makers to access, analyze and develop informative outputs. The National Evaluation Platform (NEP) is an approach to large-scale program evaluation of complex, multi-faceted MNCH&N programs [1]. A key component the original NEP concept involved developing an integrated set of data organized by geographic area that draws from a variety of data sources available in the country including household surveys (Demographic Health Surveys, Multiple Indicator Cluster Surveys, etc), facility surveys (Service Provision Assessment, etc) and administrative sources [2]. The data include core indicators from MNCH&N program impact pathways to be updated as new data are released, facilitating rapid analysis in response to time-sensitive questions from MNCH&N stakeholders [3]. In 2013, the Government of Canada funded the real-world implementation of NEP by public-sector stakeholders in four countries in sub-Saharan Africa: Malawi, Mali, Mozambique, and Tanzania. The Institute for International Programs at the Johns Hopkins Bloomberg School of Public Health (IIP) provided technical support. In early 2014, the IIP team began to explore options for operationalizing an integrated district-level data set referred to as the “NEP data system”. In this viewpoint, we describe development, implementation challenges and lessons learned from design and deployment of a data system built on District Health Information System 2 (DHIS 2) platform in four countries in sub-Saharan Africa. As the “big data” era advances and use of DHIS 2 becomes more widespread, we hope this documentation will be useful to software developers and others aiming to build data systems that support evidence-based decision-making.

## NEP DATA SYSTEMS

At the inception of the NEP project, there were several frameworks (eg, Health Metrics Network supported by World Health Organization) and software (eg, DevInfo supported by UNICEF) for large-scale data systems that integrated data into a repository [4,5]. These tools allow for pre-calculated indicators from various data sources to be housed in a single data set; however, users cannot manipulate data within the system (eg, to modify the indicator definition). For the NEP data system, we needed to allow for flexible data manipulation functions and in particular for the calculation of new indicators so that users could quickly generate and display coverage indicators. Other core requirements of the NEP data system identified at project inception included: 1) repository functionality for cleaned and quality-checked district-level data from a variety of survey and health management information system (HMIS) sources; 2) ability to capture individual-level data (eg, respondent-level data from surveys) and aggregate indicators by groupings of interest (eg, household wealth, urban/rural); 3) storage of data source documentation; 4) capacity for visualization and analysis either within the data system or after exporting it to a statistical package; 5) ease of use by public health government officials; and finally; 6) avoidance of introducing a parallel data system at country level.

## DHIS 2

At the start of the NEP project, we explored several software platforms and chose the DHIS 2. The online, open-source software is used by 47 governments and 23 organizations for reporting of routinely collected health system data [6]. Within DHIS 2, data elements are organized by geographic unit such as district or region. The data may be combined into indicators and displayed through tables, figures, or maps within the software. DHIS 2 requires relatively minimal training to use. An advantage of the open source software is that external applications (apps) can be developed to introduce new functionality to the core platform.

At project inception, the DHIS 2 appeared to meet most of the core requirements for an NEP data system. Three NEP countries were already using DHIS 2 as their national HMIS platform. This presented a unique opportunity to avoid training duplication as many NEP participants would likely be familiar with the system. This also provided a longer-term possibility of directly adding the data assembled for NEP within the country’s HMIS, thus avoiding parallel systems.

## NEP INNOVATIONS FOR DHIS 2

DHIS 2 is designed to capture count data directly entered by users, but for NEP we needed a way to import data from other sources in a way that could be manipulated to calculate new indicators and aggregate to unique sub-groups. This is particularly challenging for data from population-based surveys, many of which require individual observation data to be weighted according to the survey’s sample design. In order to add this new functionality, in 2014 we partnered with 2Paths Solutions Ltd (Vancouver, BC, Canada) for software development and BAO Systems (Washington, DC, USA) for system configuration design and web hosting. Working with these partners, we developed a new data import app and supported developments to the core DHIS 2 code.

The *NEP Import app* allows users to import two types of data: 1) individual records from household and facility surveys, and 2) data that have been pre-aggregated by a geographic unit (eg, district or region). NEP-funded core DHIS 2 developments enable users to generate indicators using sampling weights and to view data from multiple sources (eg, household surveys from different time points) in a single table or figure.

## NEP DATA SYSTEM CHALLENGES

In mid-2015, we introduced a beta version in each of the four NEP countries. The first task was for the country NEP data managers to upload relevant survey data and generate a dashboard with key indicators related to the first round evaluation questions. The data managers were trained through cross-country workshops, one-on-one mentorship, training manuals and other supporting resources. All four country teams were able to upload some first round data and create dashboards. However, by early 2016, nearly a year after first introducing the system, we found that most of the NEP country teams were not continuing to use the data system. Later that year, we conducted interviews with the in-country NEP data managers and IIP team members to identify implementation challenges and lessons learned.

We struggled, as software development does, with balancing a user-friendly interface with sufficient modifiability to meet the user needs. Users found the NEP import app difficult to navigate. Significant data pre-processing is required before import. For example, the import app matches a DHIS 2 organizational unit to geographic or facility names in data to be imported, so spellings of these character strings must be confirmed if they vary across data imports. When importing, users were frustrated by a high failure-to-success ratio and a lack of clear error messages explaining how to troubleshoot. The length of time required to complete an import varied widely depending on the data type, taking several hours for large household survey data sets. Program indicator calculations involve inputting complex conditional logic statements and modifying the calculations requires frequent clearing of the browser cache. The open source development community was limited on how to respond to these user issues given the programming constraints of the DHIS 2 software. Additionally, the DHIS 2 software is continuously being updated and modified, and the NEP import app requires corresponding updates by software developers.

Early in development we also discovered that it was not feasible to calculate standard errors within the DHIS 2 platform. Interpreting confidence intervals is a core skill for NEP users and so we explored options for linking the NEP Data System directly to a web-based version of R (an open source analytical software) that would allow more advanced statistical analysis [7]. A prototype developed by 2Paths Solutions, showed that while standard errors and other statistical test results could be imported from R into the DHIS 2 platform, there was no simple way to import the individual level survey data directly from DHIS 2 to R. 2Paths found it may be possible to create a portal that pulls data from DHIS 2 into R. However, the NEP project did not have sufficient resources to support timely development of such an application. Given that DHIS 2 is the global standard for routine data reporting systems, more work is needed on how to integrate the data within DHIS 2 with other systems. By the end of our grant we developed a data system that met many of our original criteria. However, the functionality challenges described here hindered the full implementation and use of the data system in the four countries.

**Figure:**
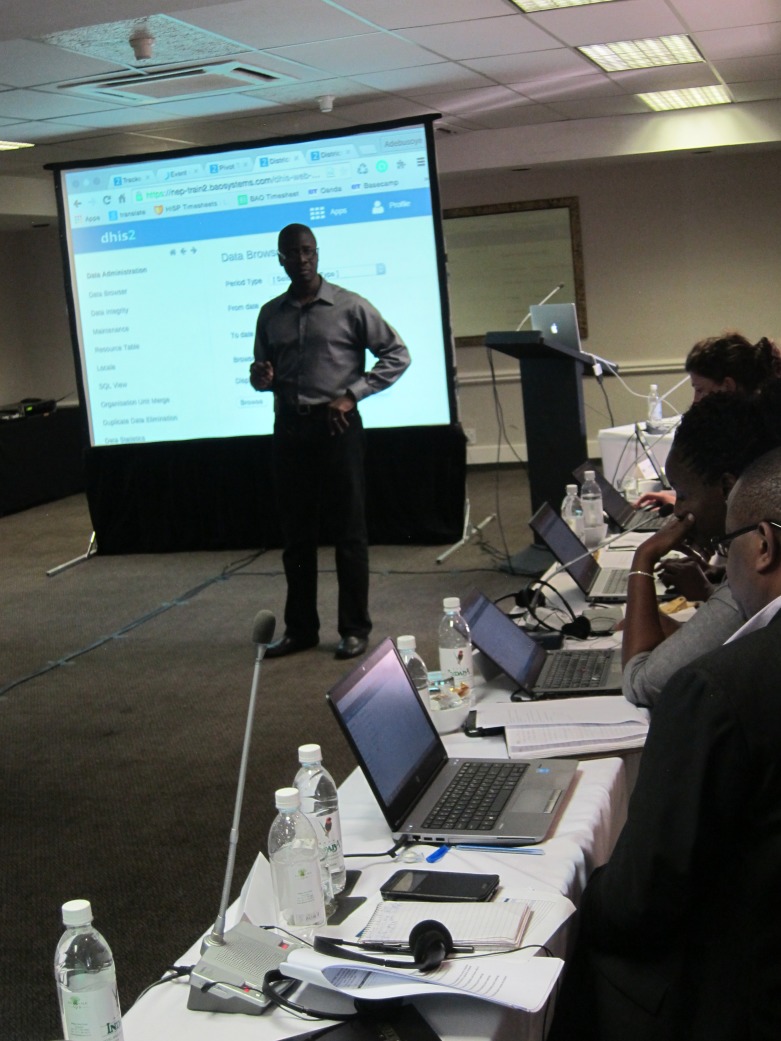
**Photo:** Busoye Anifalaje (BAO Systems) leading a DHIS 2 training in June 2015 for the NEP team (from the collection of Rebecca Heidkamp; used with permission)

## LESSONS LEARNED

NEP roll out continued successfully in the four countries in relation to overall goals [1] but unfortunately we did not have the opportunity to test whether the DHIS 2-based data system can facilitate evidence-based policy making. However, we identified lessons learned during the process that apply broadly to multi-site software development and implementation in similar contexts.

We underestimated the time needed for development and rolled-out the systems too quickly. Scaling-up to multiple country instances too early made trouble-shooting less efficient. In retrospect, we should have spent more time having our target users in a single country field test a prototype system. We tried to be efficient by hosting cross-country training but country programs were at different places in the overall project implementation. In several cases, the data managers did not have immediate tasks to work on and skills were lost.

Web-based systems that require internet connectivity are still pushing the boundaries of what the infrastructure can support in many low- and middle-income countries (LMICs). Poor internet access prevented use of this web-based platform at pivotal moments, including a demonstration during a national stakeholder meeting in Tanzania. Geographic-based data systems are also problematic. Having fixed geographic or administrative units as the system backbone makes it difficult to account for changing administrative boundaries across time.

DHIS 2 is a promising platform for building integrated data systems because it is open source and supported by a global network of software developers and implementers. We had hoped by selecting a platform already used in three of the four countries, we could reduce training time and increase use acceptability but this was not the case. In reality there is no “one-size-fits-all” software, and despite the ubiquity of DHIS 2 as a HMIS platform, it cannot fulfill all health sector monitoring and evaluation functions [8]. In retrospect, we tried to extend the functionality of DHIS 2 beyond what it was capable of doing. This is especially pertinent in the development of new features within an open-source platform. In this case the timeframe for the functionalities to mature extended well beyond the NEP project timeline.

Additionally, our original conceptualization of the data system was a data warehouse or repository model – where all data are housed in a single location to avoid duplication. Recently, another model has emerged known as “integrated health information architectures” [9]. This model acknowledges that different data, formats, and granularity are required, depending on the use case. Therefore, different data systems are needed. We began exploring this through linking DHIS 2 with R but full development of this second approach was outside the scope of the project. In retrospect, we might have further explored building a system that automatically linked in various data systems into one platform or portal for evaluation analysis rather than a data repository model.

As the availability of health-related data continues to increase in LMICs, it will become more critical for policy-makers to easily access, analysis and interpret this data. More work is needed within NEP and across the broader field of health informatics on how to organize and integrate multi-source data to better support co-analysis and ultimately produce evidence for MNCH&N program decision-making.
